# miR-145-5p Inhibits Neuroendocrine Differentiation and Tumor Growth by Regulating the SOX11/MYCN Axis in Prostate cancer

**DOI:** 10.3389/fgene.2022.790621

**Published:** 2022-03-09

**Authors:** Shuya Ji, Yi Shi, Lin Yang, Feng Zhang, Yong Li, Feng Xu

**Affiliations:** ^1^ Department of Oncology, Shanghai Pudong New Area Gongli Hospital, Shanghai, China; ^2^ Department of General Practice, Shanghai Gonghexin Road Community Health Care Service Center, Shanghai, China

**Keywords:** castration-resistant prostate cancer, neuroendocrine differentiation, miR-145-5p, SOX11, MYCN

## Abstract

Recent studies have shown that the downregulation of miR-145-5p in prostate cancer (PCa) is significantly associated with poor differentiation and prognosis. We aimed to investigate the biological role of miR-145-5p in the neuroendocrine differentiation (NED) of PCa. In this study, TheCancer Genome Atlas was used to identify the association of miR-145-5p with PCa. The functions of miR-145-5p were evaluated using the Cell Counting Kit-8 (CCK-8) assay and cell cycle analysis. We validated changes in cell cycle control by testing the expression of cyclin-related genes by western blot. The luciferase reporter assay was performed to test miR-145-5p-targeting genes and direct transcriptional targets of *SOX11*. The expression of miR-145-5p was found to be significantly downregulated in castration-resistant PCa, and this was correlated with higher Gleason score and prostate-specific antigen. We confirmed these results using PC3 and LNCaP cell lines depicted a gradual decline of miR-145-5p while the cells were cultured under androgen depletion conditions. Moreover, the knockdown of miR-145-5p significantly promoted NED and proliferation of LNCaP cells, whereas overexpression of miR-145-5p significantly inhibited NED and proliferation of LNCaP cells. Mechanistically, we found that *SOX11* was a direct target of miR-145-5p, which regulates *MYCN* might mediate induction of NED and proliferation of LNCaP cells. Furthermore, knockdown of miR-145-5p promoted tumor growth *in vivo*. Our findings suggest that miR-145-5p can inhibit NED and tumor growth by targeting *SOX11*, which regulates the expression of *MYCN*, and that this could be a novel therapeutic strategy for preventing the progression of PCa.

## Introduction

Prostate cancer (PCa) is the world’s second most common malignancy among men (Ferlay et al., 2019). In the development and progression of PCa, the androgen receptor (AR) pathway plays a key role (Giguere, 2020). For many decades, androgen deprivation therapy (ADT) has been the standard of care for patients with advanced and metastatic PCa. However, the majority of these patients develop castration-resistant PCa (CRPC) (Bishop et al., 2017). Enzalutamide (Enz) and abiraterone, two novel AR signaling inhibitors, have been approved as first-line treatments for CRPC. However, the treatment’s benefits are short-lived, and resistance develops quickly. Treatment-induced neuroendocrine PCa (NEPC) has been identified as an AR-independent resistance mechanism (Scher et al., 2012; Mu et al., 2017). Several studies have shown that neuroendocrine differentiation (NED) is correlated with tumor progression, poor prognosis, and hormone-refractory stage (Yuan et al., 2007). NEPC is associated with genomic, epigenomic, neuronal and stem cell pathway dysregulation, and epithelial-mesenchymal transition (Yamada and Beltran, 2021). However, to date, the mechanisms leading to NED in PCa progression have not been fully understood.

MicroRNAs (miRNAs) are essential epigenetic modulators in the progression of PCa. MiRNAs bind to specific sequences in the 3′-untranslated region (3′UTR) of mRNAs, causing mRNA degradation, translation, or post-transcriptional suppression, which inhibits transcript expression. Previous research has shown that miR-145-5p has a tumor-suppressing effect and is significantly downregulated in many cancers, including PCa (Avgeris et al., 2013; Xu et al., 2019; Xu et al., 2020). Several studies have used small RNA sequencing to investigate the dysregulated expression of miRNAs in CRPC and found that miR-145-5p contributes to the development of CRPC, suggesting that miR-145-5p may play a role in the NED of PCa progression (Zhu et al., 2015; Goto et al., 2017).

In this study, reduced expression of miR-145-5p was found to be strongly correlated with a higher Gleason score, N stage, and p53 mutation in PCa. In PCa cell lines, miR-145-5p levels were significantly decreased. While LNCaP cells was cultured under androgen depletion conditions, we observed a time-dependent gradual decline in the expression of miR-145-5p. According to gain- and loss-of-function studies, miR-145-5p is involved in NED and the growth of PCa cells. Mechanistically, we found that *SOX11* is a direct target of miR-145-5p, which might mediate miR-145-5p’s tumor-suppressive functions by regulating *MYCN* during NED of PCa cells.

## Materials and Methods

### Cell Lines

LNCaP and PC3 cells (Sigma-Aldrich, Hamburg, Germany) were grown in RPMI-1640 media and supplemented with 10% fetal bovine serum (FBS) or 5% charcoal-dextran-stripped FBS and penicillin-streptomycin (100 IU/ml and 100 μg/ml, respectively). All cell lines were cultured at 37°C in a humidified 5% carbon dioxide (CO2) atmosphere. Unless stated otherwise, cells were treated with 10 μM Enz (Selleck, China) for 24 h (Nguyen et al., 2014).

### Cell Transfection

Negative control (NC), miR-145-5p mimics, and inhibitor were purchased from GenePharma (Shanghai, China). Small interfering RNAs (siRNA) that specifically target human *SOX11 (si-SOX11)* and nonspecific NC oligonucleotides (si-NC) were purchased from GenePharma (Shanghai, China). According to the manufacturer’s instructions, LNCaP cells were transfected with siRNA using Lipofectamine 3000 (Invitrogen; Thermo Fisher Scientific, Inc.,, United States). Supplementary Table S1 shows the oligonucleotide sequences.

### RNA Extraction and Quantitative Real-Time Polymerase Chain Reaction

Trizol reagent (Invitrogen, Carlsbad, CA, United States) was used to extract total RNA from the cell lines according to the manufacturer’s protocol. The miRNA First-Strand cDNA Synthesis (Tailing Reaction, Sangon, China) and the PrimeScript RT Reagent Kit (Takara, Japan) were used to reverse transcribe miRNAs and mRNAs into complementary DNA (cDNA).

The ABI Prism 7500 Sequence Detection System (Applied Biosystems, Foster City, CA, United States) was used to perform quantitative PCR (qPCR) amplification using the KAPA SYBR FAST qPCR Kit. Supplementary Table S1 lists the primer sequences. For miRNAs and mRNAs, target gene expression was normalized to total U6 and actin, respectively.

### Target Prediction

The target genes of miR-145-5p were predicted using the miRSystem database (http://mirsystem.cgm.ntu.edu.tw). The miRSystem is a database that integrates DIANA, miRanda, miRBridge, PicTar, PITA, rna22, and TargetScan, which are all well-known miRNA target gene prediction programs (Lu et al., 2012). The MatInspector and JASPAR datasets were used to predict the target genes of transcription factors (Cartharius et al., 2005; Mathelier et al., 2016).

### Dual-Luciferase Assay

We constructed the *SOX11-*wild type (*SOX11*-WT) and *SOX11*-mutant (*SOX11*-Mut) 3′-UTR pmirGLO luciferase reporter vectors. HEK293T cells were seeded in 24-well plates and allowed to grow overnight before being transfected. The *SOX11*-WT or *SOX11*-Mut luciferase reporter and miRNA mimic or nonspecific NC were co-transfected into cells. Using Lipofectamine 2000 (Invitrogen; Thermo Fisher Scientific, Inc.,, MA, United States), cells were co-transfected with 0.4 µg pGL4.27-MYCN-promoter reporter plasmid and 0.3 µg pcDNA3.1-hSOX11 effector plasmid. A dual-luciferase reporter assay system was used to measure luciferase activity after incubation for 48 h according to the manufacturer’s protocol (Promega, Madison, WI, United States).

### Western Blot Analysis

Total protein was lysed from cells using radioimmunoprecipitation assay buffer (RIPA buffer; Beyotime, Shanghai, China) supplemented with protease inhibitors. SDS/PAGE was used to separate 30 µg of total proteins, which were then transferred to a nitrocellulose membrane. The membranes were blocked with 5% non-fat milk and then incubated overnight at 4°C with specific primary antibodies. The membranes were washed and incubated with secondary antibodies at room temperature for 1 h. Electro chemiluminescent detection was used to visualize the protein bands. The expressions of GAPDH and β-actin were used as internal controls. The primary antibodies were used in this study: anti-SOX11 (Santa Cruz, CA, United States), anti-MYCN (#84406, Cell Signaling Technology, United States), anti-p21 (ab109199, Abcam, United States), anti-p27 (ab32034, Abcam, United States), anti-Cyclin D1 (ab134175, Abcam, United States), anti-NSE (ab180943, Abcam, United States), anti-CHGA (ab68271, Abcam, United States), and anti-SYP (ab32127, Abcam, United States). Quantification of the western blots was performed using ImageJ.

### Cell Cycle Analysis

LNCaP-vector, LNCaP-has-miR-145-5p-mimic, and LNCaP-has-miR-145-5p-inhibitor cells transfected with *SOX11* siRNA (GenePharma, Shanghai, China) or NC were treated with Enz for 72 h. Cells were trypsinized, washed, and fixed in 70% ethanol overnight at 4°C. The following day, cells were washed, centrifuged, and stained with propidium iodide (PI) containing RNase in the dark at 37°C for 15 min. The cell cycle distribution was assessed using flow cytometry.

### Cell Proliferation Assay

According to the manufacturer’s instructions, the CCK-8 assay was used to evaluate cell proliferation (Yeasen, Shanghai, China). The experiment was performed as described previously (Mao et al., 2020).

### Mouse Xenograft Studies

Twelve 5 week-old male nude BALB/c mice were obtained from the Shanghai Sipper-BK Laboratory Animal Company (Shanghai, China). The testicles of the mice were surgically removed and split into two groups at random. At 6-weeks after cell inoculation, both groups were subcutaneously inoculated with 3 × 10^6^ PC3 cells (co-suspended with 50% matrigel) stably expressed with sh-miR-145-5p or sh-NC. Mice were sacrificed after 8 weeks, and all tumor xenografts were collected.

### Statistical Methods

Statistical analyses were performed using Prism 8 software (GraphPad Software, Inc., La Jolla, CA), and the results are presented as mean ± standard deviation. Error bars represent the normalized standard of the mean for at least three experiments. The analysis of variance was performed using the Student’s *t*-test. *p* < 0.05 was considered statistically significant.

## Results

### Decreased miR-145-5p Expression Is Associated With PCa Neuroendocrine Transdifferentiation

**FIGURE 1:**
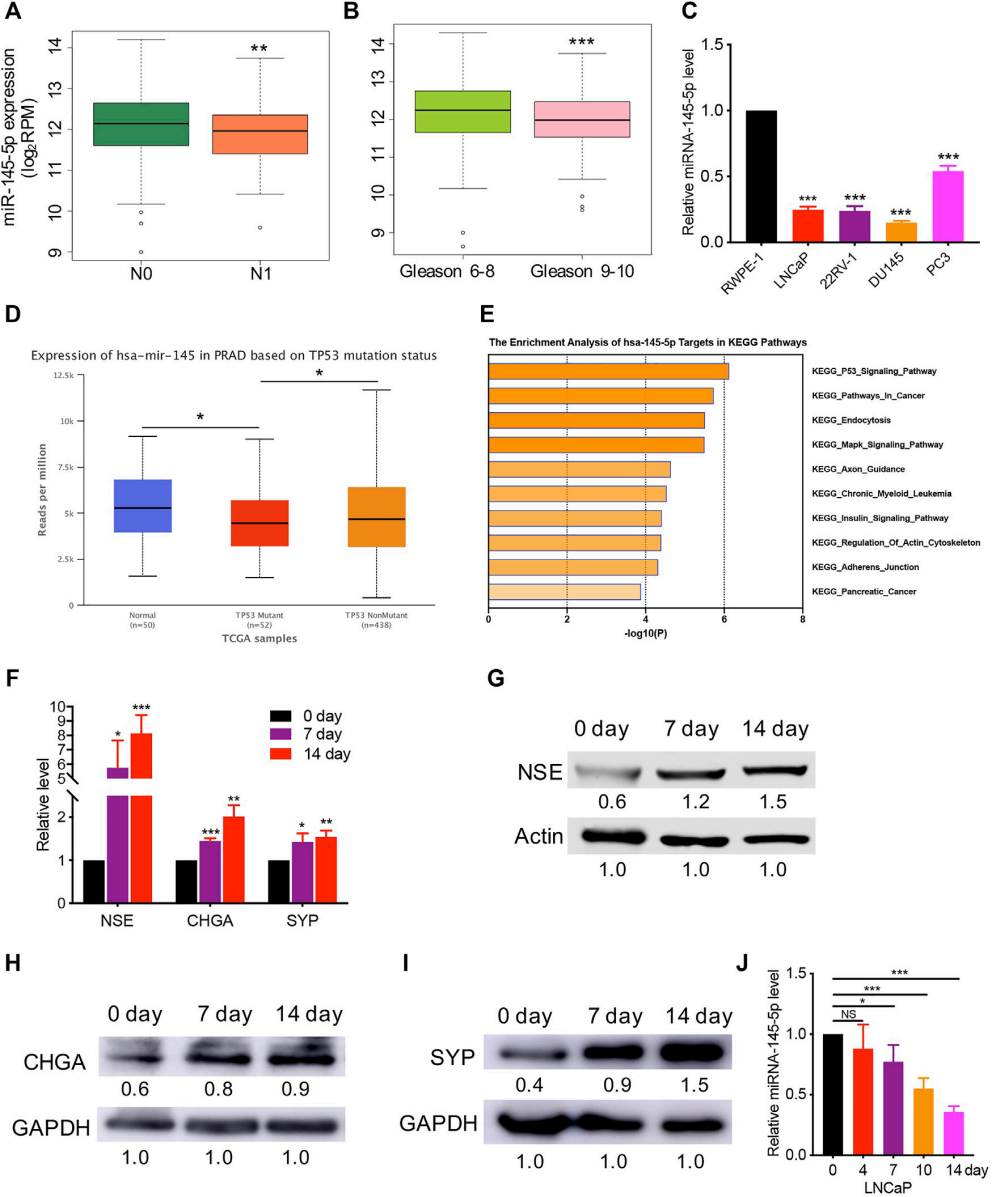
Decreased expression of miR-145-5p has correlated with prostate cancer(PCa) neuroendocrine transdifferentiation. **(A, B)** The decreased expression of miR-145-5p is correlated with lymph node metastasis and a higher Gleason score. **(C)** Quantitative real-time polymerase chain reaction (qRT-PCR) analysis of miR-145-5p expression in PCa cells compared with those in normal cells. **(D)**. Expression level of miR-145-5p in p53 mutation tissues compared to that in p53 nonmutation PCa and normal samples. **(E)** Gene set enrichment analysis of miR-145-5p targets in Kyoto Encyclopedia of Genes and Genomics pathways. **(F–I)** qRT-PCR and western blot analysis of neuroendocrine markers, including NSE, CHGA, and SYP. **(J)** Expression level of miR-145-5p during the neuroendocrine differentiation of LNCaP. The analysis was performed on PCa tumor data extracted from The Cancer Genome Atlas **(A–D)**. *p < 0.05, **p < 0.01, ***p < 0.001.

Using The Cancer Genome Atlas (TCGA) data, we discovered that lower miR-145-5p expression was linked to a higher N stage and Gleason score, which is a grading system for determining the aggressiveness of PCa (Figures 1A,B). When compared to normal prostatic epithelial cell lines such as RWPE-1, the expression of miR-145-5p is significantly downregulated in PCa cells (Figure 1C). The expression of miR-145-5p was significantly downregulated in p53 mutation than in p53 nonmutation PCa, and normal tissues (Figure 1D). Gene set enrichment analysis of miR-145-5p targets in Kyoto Encyclopedia of Genes and Genomics pathways showed that starBase online enriched the p53 signal pathway (Figure 1E). Moreover, miR-145 is important in the differentiation of human embryonic stem cells (Xu et al., 2009). This research suggests a link between decreased expression of miR-145-5p and NEPC, which could explain the biological behavior of prostatic NED. LNCaP cells, which are androgen-sensitive prostate adenocarcinoma cells, were used to test this hypothesis. After 14 days of androgen depletion, LNCaP cells displayed morphological changes, including neurite-like outgrowths and decreased cell growth (Supplementary Figure S1A). RT-qPCR and western blot analysis revealed that the neuroendocrine (NE) markers NSE, CHGA, and SYP were gradually upregulated at both the mRNA and protein levels (Figures 1F–I). However, during the NED of LNCaP cells, miR-145-5p gradually decreased (Figure 1J). These results suggest that miR-145-5p may be involved in the induction of NEPC.

### The Downregulation of miR-145-5p Expression Promotes the NED and Proliferation of PCa Cells

**FIGURE 2:**
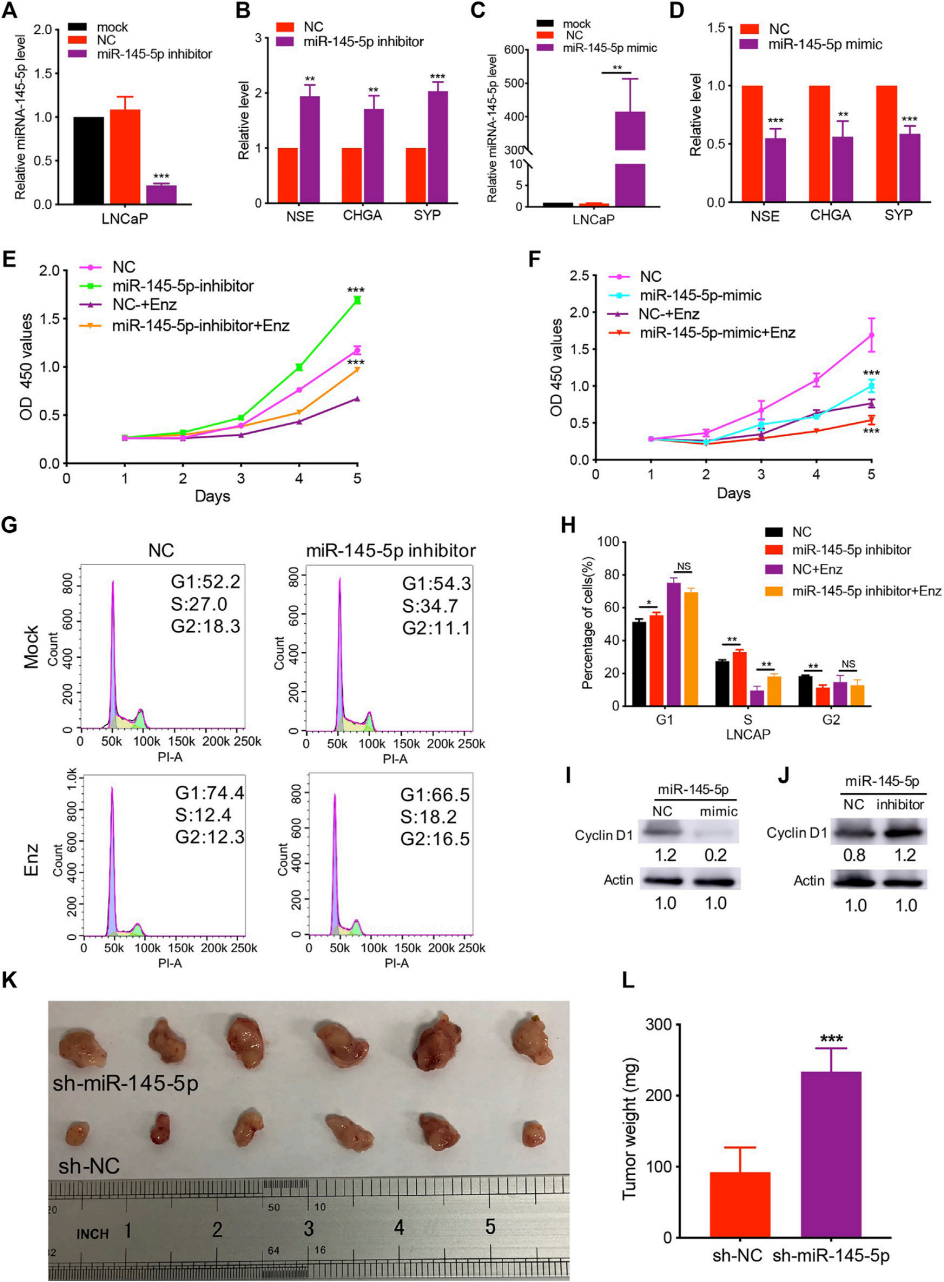
Knockdown of miR-145-5p promoted neuroendocrine differentiation and proliferation of prostate cancer (PCa) cells. **(A)** The results of quantitative real-time polymerase chain reaction (qRT-PCR) show the efficiency of miR-145-5p knockdown. **(B)** qRT-PCR analysis of neuroendocrine (NE) markers in LNCaP cells transfected with miR-145-5p inhibitor or negative control (NC). **(C)** The results of the qPCR show the efficiency of miR-145-5p overexpression. **(D)** qRT-PCR analysis of NE markers in LNCaP cells transfected with miR-145-5p mimics or NC. **(E)** The Cell Counting Kit-8 (CCK-8) proliferation assay shows the proliferation ability of miR-145-5p-knockdown LNCaP cells or control cells with or without enzalutamide (Enz) exposure. **(F)** The CCK-8 proliferation assay shows the proliferation ability of miR-145-5p-overexpressing LNCaP cells or control cells with or without Enz exposure. **(G, H)** Flow cytometry analysis of the cell cycle of miR-145-5p-knockdown LNCaP cells or control cells with or without Enz exposure. **(I)** Western blot analysis of Cyclin D1 proteins miR-145-5p-overexpressing LNCaP cells or control cells with Enz exposure. **(J)** Western blot analysis of Cyclin D1 proteins miR-145-5p-knockdown LNCaP cells or control cells with Enz exposure. **(K, L)** The results of the subcutaneous xenograft mouse model show that the knockdown of miR-145-5p promoted PCa growth *in vivo*. *p < 0.05, **p < 0.01, ***p < 0.001.

Using miRNA transfection of LNCaP cells, we performed gain- and loss-of-function studies to investigate the role of miR-145-5p on NED of PCa. Indeed, RT-qPCR revealed that downregulation of miR-145-5p promoted the upregulation of NE markers expression compared to cells transfected with NC (Figures 2A,B). In comparison to control cells, overexpression of miR-145-5p downregulated the expression of NE markers (Figures 2C,D). There was no obvious change in AR protein in LNCaP cells with the knockdown or overexpression of miR-145-5p (Supplementary Figure S1B). Furthermore, when miR-145-5p was knocked down, the proliferation of LNCaP cells increased compared to controls (Figure 2E). The proliferation assay revealed that the overexpression of miR-145-5p markedly inhibited the proliferation of LNCaP cells compared to NC (Figure 2F). To learn more about how miRNA knockdown enhances cell proliferation, we used PI staining and fluorescence-activated cell sorting analysis to look at the cell cycle distribution of transfected LNCaP cells. The proportion of cells in the S phase was significantly higher in miR-145-5p knockdown cells (Figures 2G,H), indicating that the knockdown of miR-145-5p resulted in increased cell cycle progression. Using western analysis, we discovered that overexpression of miR-145-5p decreased the expression of Cyclin D1 but increased the expressions of p21 and p27 (Figure 2I; Supplementary Figure S1C). Moreover, knocking down miR-145-5p increased the expression of Cyclin D1 while decreasing the expressions of p21 and p27 in PCa cells (Figure 2J; Supplementary Figure S1D). These findings suggested that miR-145-5p inhibited the proliferation of LNCaP cells by regulating the cell cycle progression. We constructed a stable knockdown of miR-145-5p and control cells with shRNA to further determine the effect of miR-145-5p *in vivo* (Supplementary Figure S1E). We chose PC3 with NE characteristics for the subcutaneous tumorigenesis experiment because LNCaP cells are difficult to develop into xenograft tumors, *in vivo,* under androgen depletion conditions. The results showed that the knockdown of miR-145-5p in PCa cells promoted tumor growth *in vivo* (Figures 2K,L). These findings showed that the downregulation of miR-145-5p expression promoted the NED and proliferation of PCa cells.

### *SOX11* Mediates Decreased miR-145-5p-Induced NED of LNCaP Cells

**FIGURE 3:**
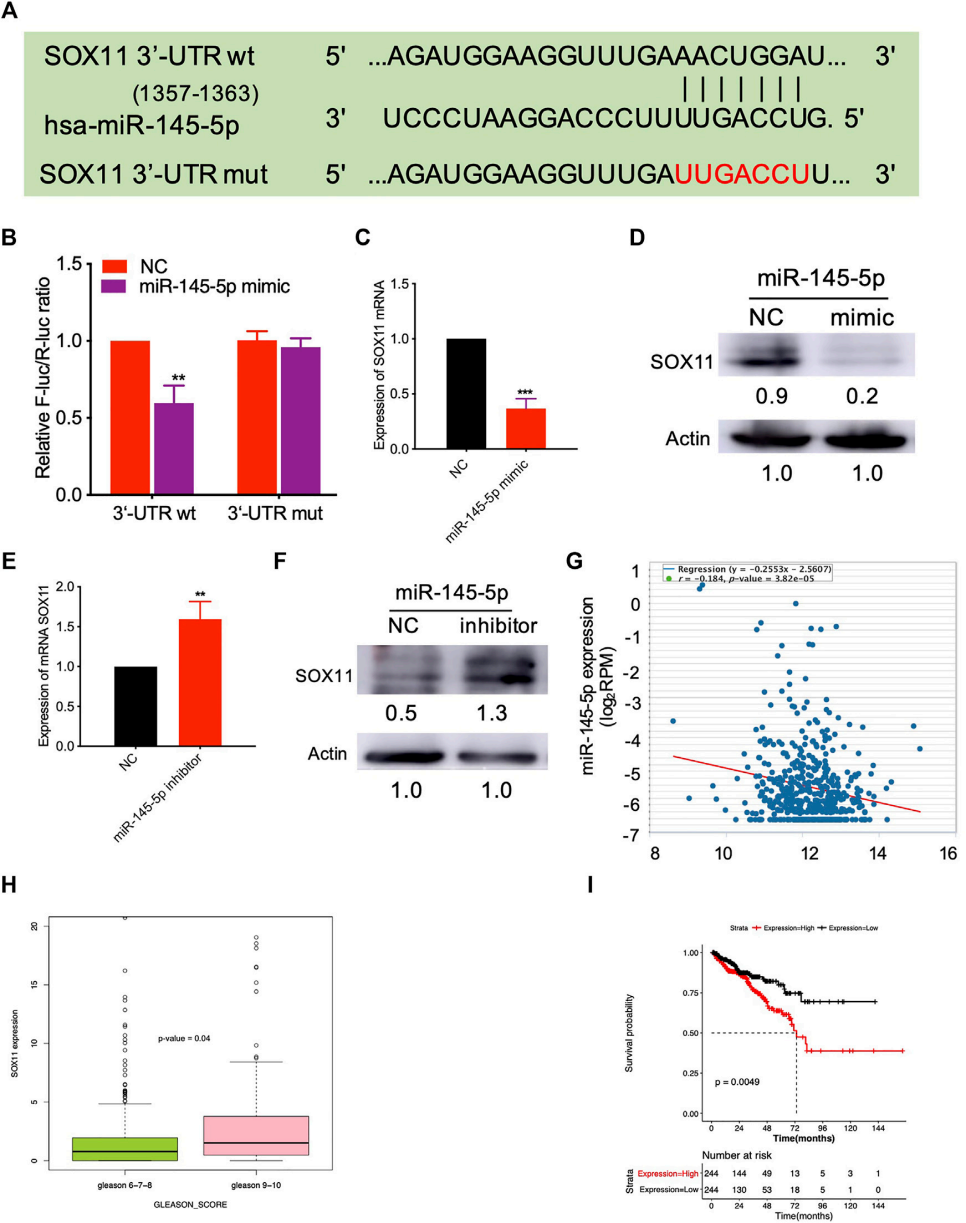
miR-145-5p directly targets *SOX11*. **(A)** Schematic exhibiting the predicted miR-145-5p interaction sites in the 3′UTR region of *SOX11*. **(B)** The luciferase reporter gene assay showed the binding of miR-145-5p to *SOX11* 3′UTR compared to the empty reporter gene vector. **(C, D)** Quantitative real-time polymerase chain reaction (qRT-PCR) and western blot analysis of SOX11 mRNA and protein levels in miR-145-5p-overexpressing LNCaP cells or control cells. **(E, F)** qRT-PCR and western blot analysis of SOX11 mRNA and protein levels in miR-145-5p-knockdown LNCaP cells or control cells. **(G)** The correlation between miR-145-5p and SOX11 mRNA in prostate cancer (PCa) in The Cancer Genome Atlas (TCGA) data by starBase. **(H)** The correlation between SOX11 mRNA and Gleason score in PCa in TCGA data. **(I)** Kaplan–Meier analysis shows the association of *SOX11* expression with disease-free survival of 488 patients with PCa from TCGA data. *p < 0.05, **p < 0.01, ***p < 0.001.

We predicted the targets of miR-145-5p using miRSystem and selected *SOX11* as a potential miR-145-5p target gene (Supplementary Table S2). Figure 3A illustrates the predicted miRNA interaction sites within the 3′UTR region of *SOX11*. The *SOX11* 3′UTR fragment containing the putative miRNA target site was incorporated into a luciferase reporter vector. *SOX11* luciferase vector and miRNA-145-5p mimics were co-transfected into HEK293T cells. When compared to NC, miR-145-5p significantly reduced the activity of the luciferase reporter gene (*p* < 0.05) under the regulatory control of the *SOX11* 3′UTR fragment (Figure 3B). SOX11 mRNA and protein levels were reduced as a result of the upregulation of miR-145-5p (Figures 3C,D). Furthermore, knocking down miR-145-5p could upregulate SOX11 mRNA and protein levels (Figures 3E,F). Using TCGA data, we validated our *in vitro* findings in a cohort of patients with PCa. In PCa, miR-145-5p has a negative correlation with SOX11 mRNA (Figure 3G). Furthermore, increased expression of *SOX11*is correlated to a higher N stage (Figure 3H). Subsequently, we performed a Kaplan–Meier analysis on 488 patients and found that higher expression of *SOX11* was associated with shorter disease-free survival (DFS) (log-rank test, *p* = 0.0049, Figure 3I).

**FIGURE 4:**
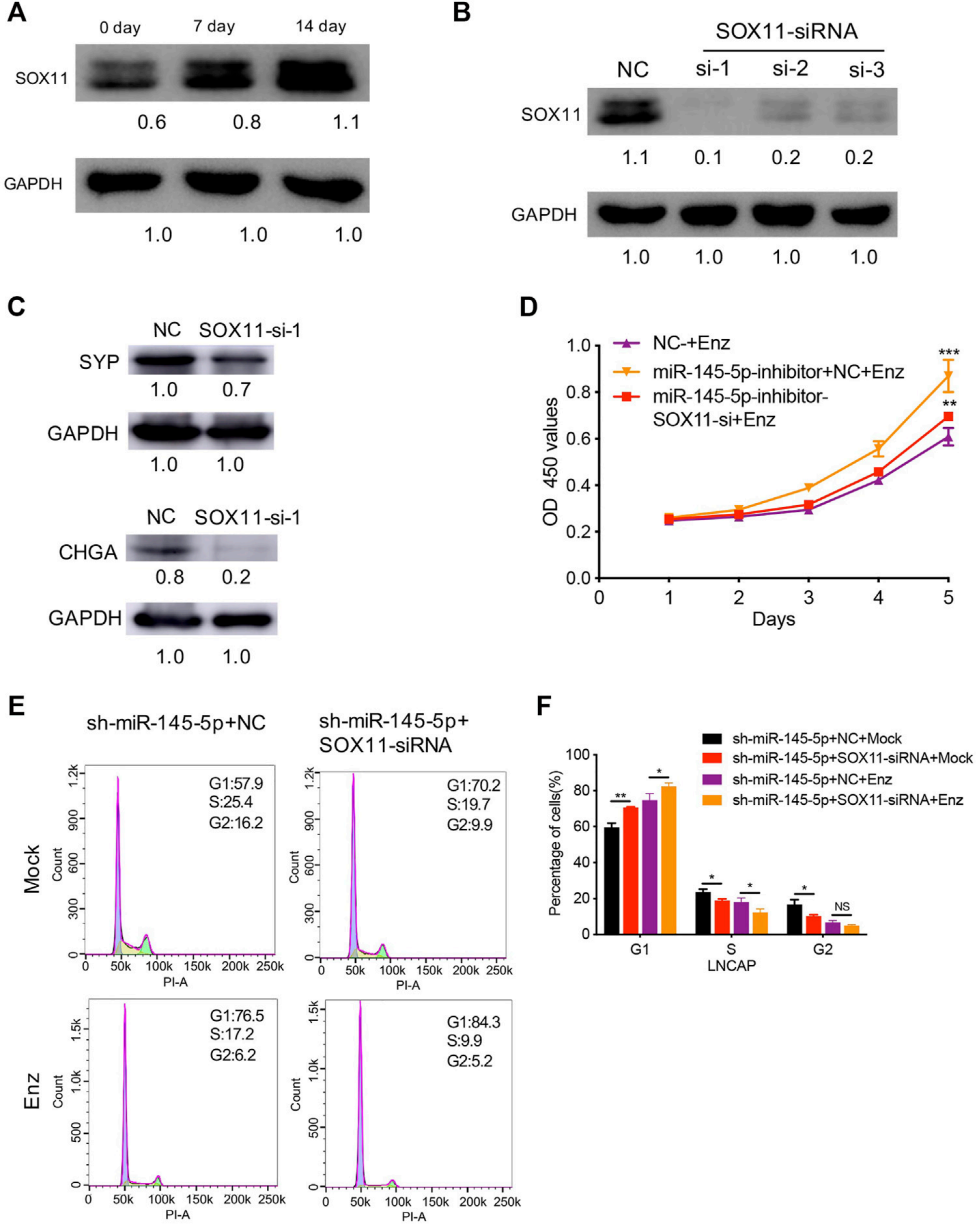
*SOX11* mediates a decrease in miR-145-5p-induced neuroendocrine differentiation (NED) of LNCaP cells. **(A)** Western blot analysis of *SOX11* protein level during the NED of LNCaP. **(B)** The results of the western blot show the efficiency of *SOX11* knockdown. **(C)** Western blot analysis of neuroendocrine markers in *SOX11*-knockdown LNCaP cells or control cells upon enzalutamide (Enz) exposure. **(D)**. The Cell Counting Kit-8 proliferation assay shows the proliferation ability of miR-145-5p-knockdown LNCaP cells with *SOX11* knockdown upon Enz exposure. **(E, F)** Flow cytometry analysis showed that compared with miR-145-5p-knockdown cells, the proportion of S phase cells was significantly reduced in the double knockdown of miR-145-5p and SOX11 with or without Enz exposure. *p < 0.05, **p < 0.01, ***p < 0.001.

Next, using RT-qPCR and western blot analysis, we discovered increased *SOX11* expression associated with NED of LNCaP (Figure 4A). In comparison to control cells, knockdown of *SOX11* inhibited the expression of NE markers (Figures 4B,C). Then, with a miR-145-5p knockdown *SOX11* knockdown rescued the sensitivity of LNCaP cells to Enz, resulting in the decreased proliferation of LNCaP cells compared to controls (Figure 4D). Compared with only miR-145-5p-knockdown cells, the proportion of S phase cells was significantly reduced in the double knockdown of miR-145-5p and *SOX11* with or without Enz exposure (Figures 4E,F), indicating that *SOX11* mediates knockdown of miR-145-5p and thus promotes cell cycle progression. Together, these findings show that the downregulation of miR-145-5p expression promotes the NED and proliferation of PCa cells by directly upregulating *SOX11*.

### miR-145-5p Negatively Regulates *MYCN* by Repressing *SOX11* Expression in NED

**FIGURE 5:**
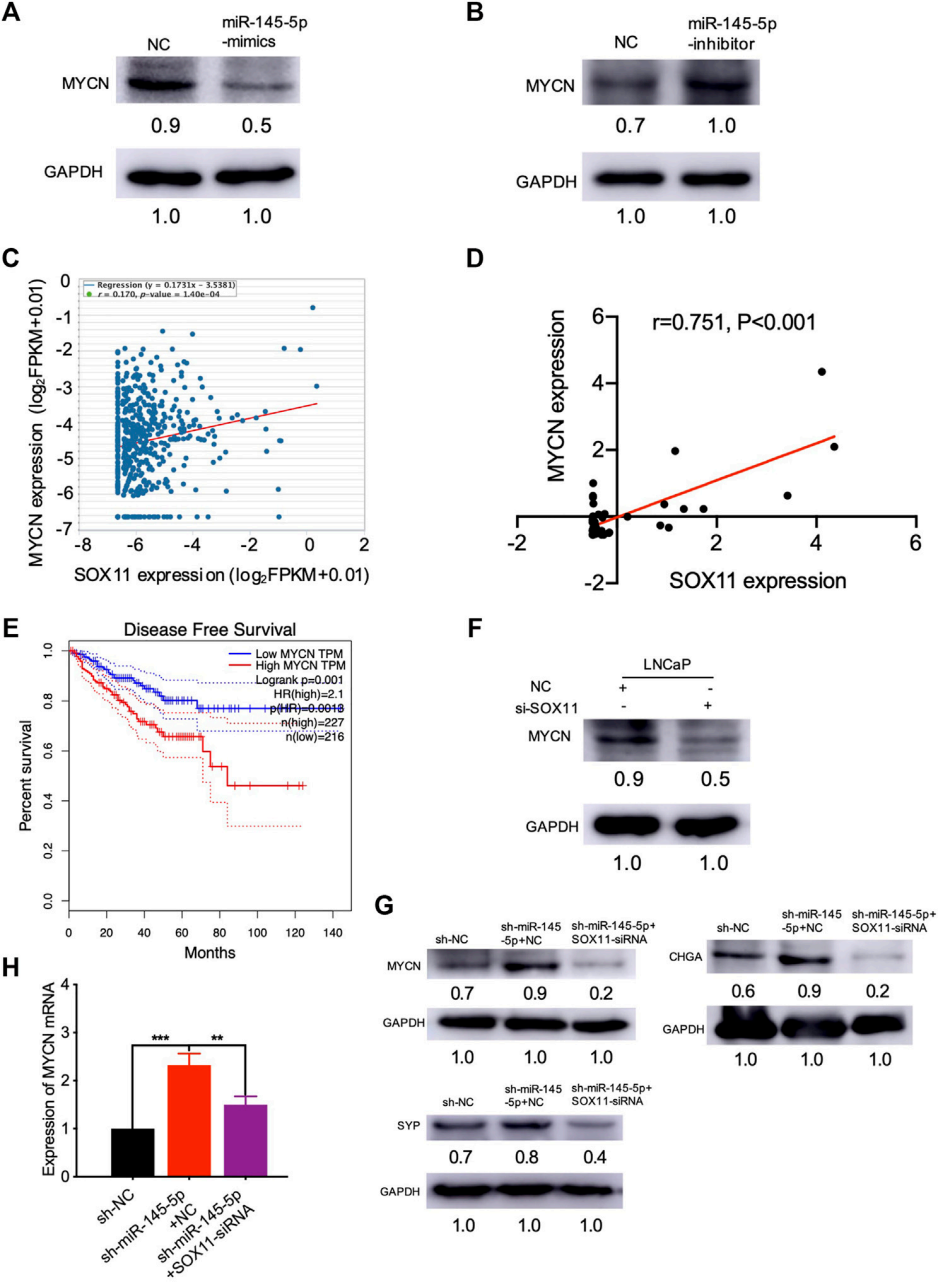
miR-145-5p negatively regulates *MYCN* by repressing the expression of *SOX11* in neuroendocrine differentiation. **(A)** Western blot analysis of *MYCN* protein in miR-145-5p-overexpressing LNCaP cells or control cells upon enzalutamide (Enz) exposure. **(B)** Western blot analysis of *MYCN* protein in miR-145-5p-knockdown LNCaP cells or control cells upon Enz exposure. **(C)** The correlation between *SOX11* mRNA and *MYCN* expression in prostate cancer (PCa) in The Cancer Genome Atlas (TCGA) data by starBase. **(D)** The correlation between *SOX11* mRNA and *MYCN* expression in the castration-resistant prostate cancer subgroup in TCGA data. **(E)** Kaplan–Meier analysis shows the association of *MYCN* expression with disease-free survival of 488 patients with PCa from TCGA data by gene expression profiling interactive analysis. **(F)** Western blot analysis of *MYCN* protein in *SOX11*-knockdown LNCaP cells or control cells upon Enz exposure. **(G, H)** Western blot analysis and the quantitative real-time polymerase chain reaction of *MYCN* protein and neuroendocrine markers of miR-145-5p-knockdown and the double knockdown of miR-145-5p and *SOX11* LNCaP cells. *p < 0.05, **p < 0.01, ***p < 0.001.

We were interested in identifying which mediators are responsible for NED after *SOX11* is knocked out. Using the MatInspector and JASPAR datasets, multiple studies have revealed the *SOX11* targets (Cartharius et al., 2005; Mathelier et al., 2016). In contrast to controls, we found that overexpression of miR-145-5p repressed the expression of *MYCN* in PCa cell lines (Figure 5A). Knockdown of miR-145-5p, on the other hand, increased the expression of *MYCN* (Figure 5B). PCa samples from TCGA show that *MYCN* was positively correlated with SOX11 mRNA levels (Figure 5C). Furthermore, *MYCN* was significantly correlated with the expression of *SOX11* in the CRPC subgroup (Figure 5D) and shorter DFS (Figure 5E). Western blot analysis confirmed that *SOX11* knockdown decreased the expression of *MYCN* (Figure 5F). The double knockdown experiment showed that *SOX11* regulates *MYCN* through miR-145-5p to induce NED (Figures 5G,H). However, the luciferase reporter gene experiment showed that *SOX11* did not activate the expression of *MYCN* 2000 bp upstream of the *MYCN* promoter (Supplementary Figure S1F). These findings demonstrated that decreased miR-145-5p expression resulted in increased expression of *SOX11* and *MYCN,* promoting the NED and proliferation of PCa cells.

## Discussion

The histologic subtype of PCa known as NEPC is extremely aggressive. After treatment with ADT, PCa cells exhibit lineage plasticity, which is a common change in cellular phenotypes (Mu et al., 2017). The emergence and maintenance of NEPC have been linked to many molecular mechanisms, including gene mutations, apparent changes, transcription factors, and other pathways. Genomic alterations in *TP53, Rb1, PTEN,* and *MYCN* have been shown to play a critical role in the development of NEPC (Ge et al., 2020). Apart from these alterations, epigenetic changes, including the aberrant expression of EZH2, CBX2, and HOTAIR have been identified as key drivers of NEPC. Several transcription factors, including BRN2, SOX2, FOXA1, and REST, have been shown to enhance or repress the neuroendocrine lineage phenotype. Other regulators directly or indirectly involved in cell lineage plasticity, such as PRKCI, SRRM4, and AURKA, have been reported in NEPC in addition to genomic alterations, epigenetic regulators, and transcription factors (Wang et al., 2021). miR-145-5p is a well-studied tumor suppressor miRNA that is downregulated in many human cancers, including breast, bladder, and prostate cancer (Ye et al., 2019). Downregulation of miR-145 occurs as a result of DNA methylation and p53 mutation in PCa, suggesting that miR-145-5p might play an important role in the initiation and progression of PCa (Suh et al., 2011). According to emerging evidence, decreased miR-145-5p expression has been linked to the development of mCRPC and a shorter DFS in PCa (Avgeris et al., 2013; Zhu et al., 2015; Goto et al., 2017). Furthermore, by targeting pluripotency factors like OCT4, SOX2, and KLF4 in various cancers, miR-145-5p has been shown to suppress the cancer stem cell-like properties and contribute to chemotherapy and radiation sensitivity (Ye et al., 2019). As a result, the goal of this study was to identify the role and molecular mechanism of miR-145-5p in the development of NEPC.

Next, we discovered that miR-145-5p targets were correlated with the p53 mutation involved in NEPC. Furthermore, we found that miR-145-5p levels gradually decreased as LNCaP cells underwent NE transdifferentiation. Moreover, knockdown and overexpression of miR-145-5p promoted the upregulation and downregulation of NE markers, NSE, CHGA, and SYP. The proliferation assay revealed that downregulating miR-145-5p increased the proliferation of LNCaP cells while overexpressing miR-145-5p significantly decreased the proliferation of LNCaP cells. Subsequently, we found that knocking down miR-145-5p in PCa cells increased Cyclin E1 and Cyclin D1 while decreasing p21 and p27 expressions, explaining the increased cell cycle progression. Together, these findings demonstrated that decreased miR-145-5p expression promoted NED and proliferation of PCa cells.

miR-145-5p has been found to suppress cancer cell proliferation, invasion, stemness, chemotherapy, and radiation sensitivity in patients with PCa by targeting different regulators such as DNMT3a, TWIST1, ITPR2, AR, and NMT3b (Larne et al., 2015; Wang et al., 2015; Xue et al., 2015; Rajabi et al., 2020; Huang and Tang, 2021). Additionally, circular RNAs (circRNAs) are a novel class of miR-145-5p target genes, according to a recent study using circRNA array analysis of LNCaP cells overexpressing miR-145 (He et al., 2018). According to bioinformatics and correlation analysis, *SOX11* is a downstream target gene of miR-145-5p in PCa. Recent studies also point out that miR-145-5p inhibits cell proliferation and induces cell apoptosis in the bladder and endometrial cancer cells by targeting *SOX11* (Chang et al., 2017; Wu et al., 2018). *SOX11* is a key modulator of NED in *TP53*-deficient CRPC (Zou et al., 2017). Coincidentally, this study also confirmed that decreased miR-145-5p expression directly upregulated *SOX11* to promote the NED and proliferation of PCa cells.

We also found that miR-145-5p overexpression suppressed the expression of *MYCN* in PCa. The induction of the NEPC histological phenotype was correlated to the overexpression of *MYCN* (Beltran et al., 2011; Lee et al., 2016). By binding to AR enhancers, *MYCN* functions to inhibit AR signaling and drives NEPC in human prostate epithelial cells (Dardenne et al., 2016; Lee et al., 2016). In the TCGA database, we found that *SOX11* was significantly correlated with the expression of *MYCN* in the CRPC subgroup. The double knockdown experiment showed that *SOX11* regulates *MYNC* to induce NE transdifferentiation, and thus mediates miR-145-5p. However, the luciferase reporter gene experiment showed that *SOX11* did not activate the expression of *MYCN*, suggesting that *SOX11* binds to sites other than the promoter region to directly or indirectly regulate the transcription of *MYCN*. These findings suggest that downregulation of miR-145-5p expression resulted in the increased expression of *SOX11* and *MYCN,* promoting the NED and proliferation of PCa cells.

In the present study, we found that miR-145-5p decreased gradually during androgen depletion, but the mechanism of its upstream regulation needs to be investigated further. AR was found to suppress the expression of miR-145 in renal cell carcinoma by directly binding to the AR element located on the promoter region of miR-145 and suppressing p53’s induction of miR-145 (Chen et al., 2015). Previous research suggested that p53 could activate the miR-145 promoter by binding to the p53 response element (p53RE) (Sachdeva et al., 2009). According to recent studies, loss of *TP53* and *RB1* function confers antiandrogen resistance by inducing NED (Ku et al., 2017; Mu et al., 2017). As a result, we speculate that as ADT screening pressure increases, the proportion of cells with the p53 mutation gradually increases, failing to relieve AR-mediated transcriptional repression of miR-145, resulting in miR-145 decline. Additionally, in PCa, miR-145 has been implicated as a direct regulator of AR (Larne et al., 2015). These findings suggest that miR-145 may be involved in a negative feedback loop with AR signaling in PCa progression. However, when miR-145-5p was knocked down or overexpressed in LNCaP cells, there was no obvious change in the AR protein.

In conclusion, in this study, we found that decreased miR-145-5p expression is associated with PCa neuroendocrine transdifferentiation. Furthermore, we demonstrated that miR-145-5p inhibits the expression of *MYCN* by directly binding to *SOX11*, which prevents NED and inhibits the cell cycle progression of PCa cells. Our findings add to the growing body of evidence that miR-145-5p is a potential therapeutic target for NEPC in PCa, this is consistent with the report of Iscaife et al., who found that *in vivo* the therapeutic activity of the tumor suppressor miR-145 in treating metabolic PCa (Iscaife et al., 2018). More research is needed to prove its safety profile before it can be used in humans. Nevertheless, future research into the clinical significance of miR-145 in the treatment of patients with NEPC is needed.

## Data Availability

The original contributions presented in the study are included in the article/Supplementary Material, further inquiries can be directed to the corresponding authors.
