# Supplementary figures and images for: miR-145-5p Inhibits Neuroendocrine Differentiation and Tumor Growth by Regulating the SOX11/MYCN Axis in Prostate cancer
Source: Front Genet. 2022 Mar 9;13:790621. doi: 10.3389/fgene.2022.790621 (PMC8965462; doi:10.3389/fgene.2022.790621)

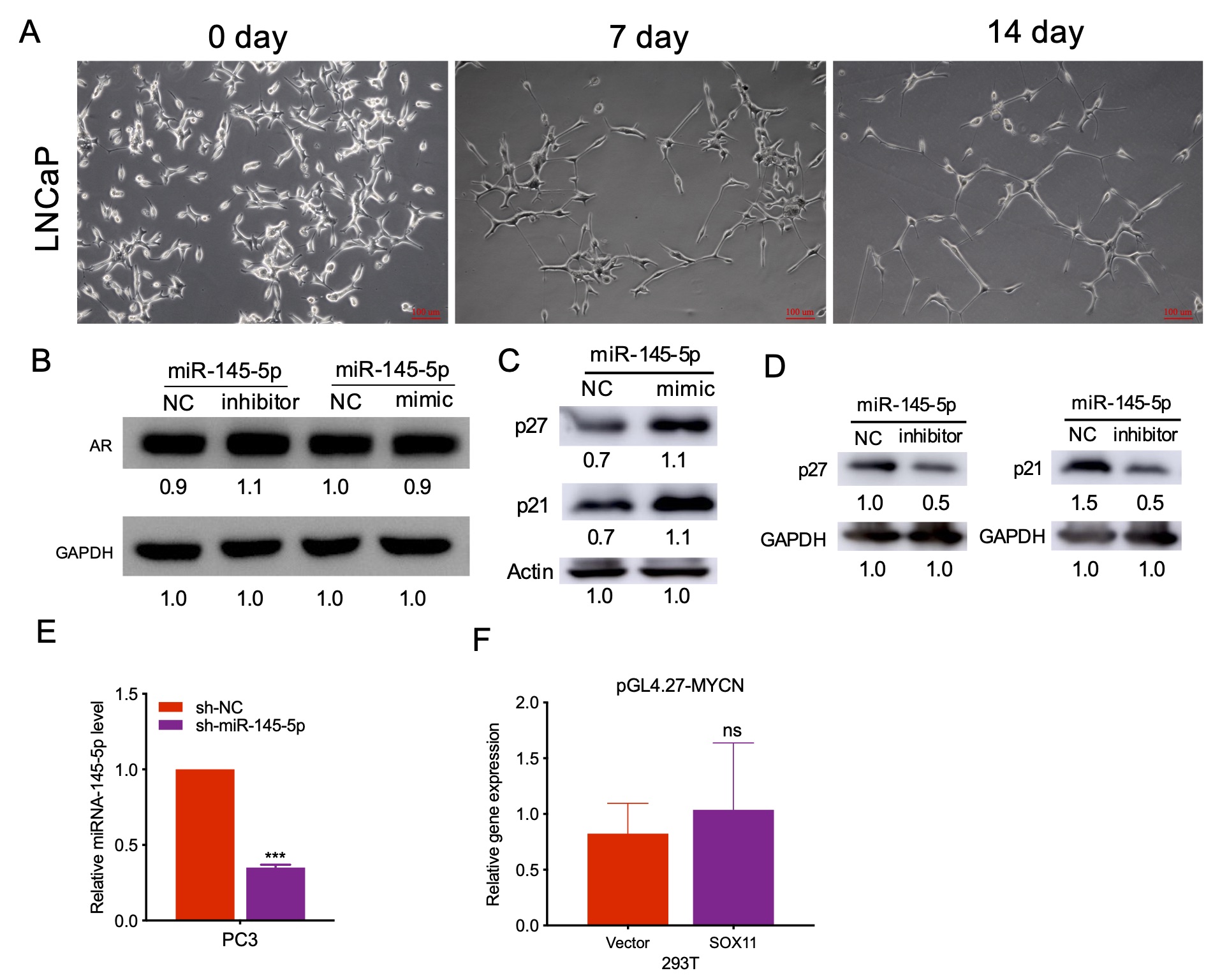

Supplement: Supplementary file 2 [file Image1.JPEG]
